# Specification of Region-Specific Neurons Including Forebrain Glutamatergic Neurons from Human Induced Pluripotent Stem Cells

**DOI:** 10.1371/journal.pone.0011853

**Published:** 2010-07-29

**Authors:** Hui Zeng, Min Guo, Kristen Martins-Taylor, Xiaofang Wang, Zheng Zhang, Jung Woo Park, Shuning Zhan, Mark S. Kronenberg, Alexander Lichtler, Hui-Xia Liu, Fang-Ping Chen, Lixia Yue, Xue-Jun Li, Ren-He Xu

**Affiliations:** 1 Department of Genetics and Developmental Biology, University of Connecticut Stem Cell Institute, University of Connecticut Health Center, Farmington, Connecticut, United States of America; 2 Department of Neuroscience, University of Connecticut Stem Cell Institute, University of Connecticut Health Center, Farmington, Connecticut, United States of America; 3 Department of Hematology, Central South University Xiang-Ya Hospital, Changsha, Hunan, China; 4 Department of Geriatrics, Central South University Xiang-Ya Hospital, Changsha, Hunan, China; 5 Department of Cell Biology, University of Connecticut Health Center, Farmington, Connecticut, United States of America; 6 Department of Regenerative Sciences, University of Connecticut Health Center, Farmington, Connecticut, United States of America; University of Washington, United States of America

## Abstract

**Background:**

Directed differentiation of human induced pluripotent stem cells (hiPSC) into functional, region-specific neural cells is a key step to realizing their therapeutic promise to treat various neural disorders, which awaits detailed elucidation.

**Methodology/Principal Findings:**

We analyzed neural differentiation from various hiPSC lines generated by others and ourselves. Although heterogeneity in efficiency of neuroepithelial (NE) cell differentiation was observed among different hiPSC lines, the NE differentiation process resembles that from human embryonic stem cells (hESC) in morphology, timing, transcriptional profile, and requirement for FGF signaling. NE cells differentiated from hiPSC, like those from hESC, can also form rostral phenotypes by default, and form the midbrain or spinal progenitors upon caudalization by morphogens. The rostrocaudal neural progenitors can further mature to develop forebrain glutamatergic projection neurons, midbrain dopaminergic neurons, and spinal motor neurons, respectively. Typical ion channels and action potentials were recorded in the hiPSC-derived neurons.

**Conclusions/Significance:**

Our results demonstrate that hiPSC, regardless of how they were derived, can differentiate into a spectrum of rostrocaudal neurons with functionality, which supports the considerable value of hiPSC for study and treatment of patient-specific neural disorders.

## Introduction

Embryonic stem (ES) cells have been derived from mouse, monkey, human, and many other species, and considered as potent candidates for regenerative medicine, and unique tools for understanding of disease mechanisms and screening for effective and safe drugs[1]. The key step toward their application in neurological diseases is to direct human ES cell (hESC) differentiation to the neural lineages and then to specific neuronal types that are affected under certain pathological conditions[2]. Since the seminal reports on neural differentiation in 2001[3], efficient neural differentiation has been achieved using several systems involving adherent culture[4], embryoid body (EB) formation[3], [5], and/or co-culture with stromal cells[6], [7]. Neurogenesis occurs when bone morphogenetic protein (BMP) signaling is inhibited, as we[8] and others[9], [10] first demonstrated in the *Xenopus* embryo, and/or when fibroblast growth factor (FGF) signaling is activated[11]. Recently, it was reported that FGF alone promotes neural differentiation from hESC, independently of BMP signaling[12].

During development, specific neural progenitors are induced along anterior-posterior (or rostral-caudal) and dorsal-ventral axes by secreted morphogens[13], [14]. Currently, protocols for generating neuronal subtypes have been developed largely based on the positional information of these cell types *in vivo*. The utilization of the morphogens such as sonic hedgehog (SHH) plus retinoic acid (RA) or SHH plus FGF8 has made it possible to produce spinal motor neurons and midbrain dopaminergic neurons, respectively, from hESC[15], [16], [17], [18], [19], [20]. Recently, we have also revealed that forebrain glutamatergic and GABAnergic neurons can be specified from hESC-derived neuroepithelial (NE) cells via modulation of WNT and SHH pathways[21]. Although hESC-derived neurons provide an important tool for studying neural genetic disorders and producing therapeutic cell types for their treatment, these applications are only possible after the difficulties of genetically manipulating hESC to model the diseases and the problem of immunorejection of hESC-derived cells by potential recipients are overcome.

The breakthroughs in generation of induced pluripotent stem cell (iPSC) via somatic cell reprogramming[22], [23], [24], [25] have made it possible to obtain human iPSC (hiPSC) from patients such as those with Parkinson disease[26] and amyotrophic lateral sclerosis[27]. These cells have the same genetic background as the patients, thus possessing tremendous potential to model the neurological diseases and generate patient-specific neurons for autogenous transplantation[28], [29]. hESC[30] and hiPSC[22], [23] are derived from totally different tissues and via different methods. They have been demonstrated to possess quite different gene expression profiles, despite great similarities in the expression patterns of pluripotency and developmental genes between both cell types[31]. Thus, it is very important to examine whether hiPSC have the same capacity to generate the whole spectrum of region-specific neural progenitors and then functional neuronal subtypes. Here, we demonstrate the efficient patterning of hiPSC-derived NE cells to region-specific progenitors along the anterior-posterior axis, which can further differentiate into functional neurons including forebrain glutamatergic neurons. Different hiPSC lines showed marked variations in the generation of NE cells, suggesting that intrinsic differences between hiPSC lines are in play.

## Methods

### Ethics Statement

All animal work was conducted according to relevant national and international guidelines (see details under the section of “hiPSC Generation”).

### Reagents

Primary antibodies used in this study included mouse antibodies against SSEA3 and TRA-1-60 (Santa Cruz Biotechnology, Santa Cruz, CA). Mouse anti-PAX6 (final dilution 1∶5000), rat anti-HOXB4 (1∶20), mouse anti-MNR2 (HB9, 1∶50), and rabbit anti-βIII-tubulin (1∶5000) antibodies were from Developmental Studies Hybridoma Bank (Iowa City, IA). Goat anti-OTX2 (1∶2000, R&D Systems Inc., Minneapolis, MN), rabbit anti-TBR1(1∶2000), and mouse anti-MAP2 (1∶2000) were from Chemicon (Billerica, MA), rabbit anti-FOXG1 (1∶100), mouse anti-S100β1:1̃, and rat anti-CTIP2 (1∶2000) from Abcam (Cambridge, MA), rabbit anti-VGLUT1 (1∶1000) from SYSY (Germany) and rabbit anti-tyrosine hydroxylase (TH) (1∶400) from Pel-Freez (Rogers, AK), and rabbit anti-Synapsin-I (1∶250) was from Calbiochem (Gibbstown, NJ). The inhibitor of FGF receptors SU5402 was from Pharmacia & Upjohn Co. (Bridgewater, NJ).

### hiPSC Generation

Human iPSC lines were established using the published protocols[22], [23], [25]. The self-inactivating lentiviral (SIN) vectors contained paired genes for reprogramming factors OCT4 and SOX2, NANOG and LIN28 or c-MYC and KLF4, and each gene pair was separated by an internal ribosome entry site for co-expression driven by the EF1α promoter. These lentiviral vectors were used as positive controls in hiPSC derivation via episomal expression of the reprogramming factors[25]. The pMXs retroviral vectors containing OCT4, SOX2, c-MYC, and KLF4 were from Addgene (Cambridge, MA). Lentiviral vector supernatants were produced by co-transfection of each lentiviral vector, pMD2.G, and psPAX2 (Addgene) into 293FT cells (Invitrogen). Retroviral vector supernatants were produced by co-transfection of each retroviral vector, pMD2.G, and Gag-pol into 293FT cells (Invitrogen). Human fetal lung fibroblast line IMR-90 purchased from ATCC (Manassas, VA) or human dermal fibroblast line HDFa from Invitrogen were seeded at 2×10^4^ cells/cm^2^ or ∼10^6^ cells/10-cm dish (56.7 cm^2^) in DMEM (Invitrogen) supplemented with 10% FBS and 0.1 mM Non-essential Amino Acids. 10-30 colonies with morphology similar to that of hESC colonies were observed per plate. Most of the potential colonies were picked up and split onto mouse embryonic fibroblast (MEF) feeder cells to derive hiPSC lines.

The resultant hiPSC lines were positive for TRA-1-60 and SSEA4 by immunofluorescence (Figure S1A) and capable of teratoma formation (Figure S1B). Silencing of the transgenes in the hiPSC lines was confirmed (Figure S1C). Teratomas were usually formed in immunocompromised SCID-beige mice about 6 weeks after intramuscular injection of 0.05 ml hiPSC suspension into a hind limb. The animals were euthanized and the tumors dissected for necropsy analysis. The animal would be also euthanized in case of any of the three conditions: (1) a bump (tumor) exceeds one cm in diameter; (2) there is any ulceration of tumors; and (3) no tumors formed within 20 weeks post-hiPSC injection. The animal use protocol was approved by the Institutional Animal Care and Use Committee according to the guidelines of the Association for the Assessment and Accreditation of Laboratory Animal Care International.

### Cell Culture

hESC lines H9 [30] and CT2 [32], [33], hiPSC lines TZ1 (generated by using the lentiviral vectors), YZ1 and YK26 (generated by using the retroviral vectors) were cultured on irradiated mouse embryonic fibroblast (MEF) cells in hESC medium, i.e., DMEM/F12 containing 20% KnockOut Serum Replacer, 0.1 mM Non-essential Amino Acids, 1 mM L-glutamine (all from Invitrogen), and 0.1 mM β-mercaptoethanol (Sigma-Aldrich) and then supplemented with 4 ng/ml bFGF (Millipore)[34]. We also used hFIB2 hiPSC line (courtesy of George Daley)[35] generated the same way as that for YZ1 and YK26.

### Induction of Region-specific Neural Cells

For generation of region-specific neural cells from hiPSC, we used protocols developed for the same purpose on hESC[16], [18], [21]. In brief, colonies of hiPSC (and hESC as positive control) were detached from feeder cells (at day 0) and suspended in hESC medium (without bFGF) for 4 days. Then these hiPSC/hESC aggregates were cultured in a neural medium consisting of DMEM/F12 (Invitrogen), N2 supplement, and 2 µg/ml heparin (Sigma-Aldrich, St. Louis, WA) without growth factors. After adherence to a plastic surface on day 6, primitive neuroepithelial (NE) cells were observed at days 8–10, followed by treatment with or without various morphogens starting to induce region-specific neural cells as detailed below. All the cells further differentiated into the definite neural epithelial cells at days 14–17, and these neural progenitor cells were plated onto ornithine/laminin-coated coverslips at day 24 for terminal differentiation.

For induction of the forebrain neural cells, a serum-free culture condition free of known morphogens was used to generate NE cells, which uniformly expressed anterior transcription factors such as OTX2, LHX2 starting at day 24, but were negative for posterior HOX proteins. For midbrain induction, the NE cells were treated with 50 ng/ml FGF8 and 100 ng/ml SHH (R&D Systems Inc., Minneapolis, MN) for one week starting at day 10. For hindbrain and motor neuron induction, hESC/hiPSC-derived NE cells were first treated with 0.1 µM RA for caudalization in the neural medium at day 10. The NE cells in the center of colonies formed neural tube-like rosettes and attached loosely to the Petri dish, whereas the peripheral flat cells adhered to the dish tightly. At day 17, the cells in the center of the colonies were gently blown off with a 5-ml serological pipette. The flat cells on the periphery remained attached.

After isolation, cell clumps of the RA group were suspended in the same neural medium in the presence of 0.1 µM RA and 100–200 ng/ml SHH, and cell clumps of the FGF8 group were suspended in the presence of 10 ng/ml FGF8. The cell clumps were cultured for one week (until day 24). After that, the NE clumps were replated on poly-ornithine/laminin coated coverslips for terminal differentiation in the presence of neural basal medium supplemented with N2 and B27. Trophic factors each at 10 ng/ml (all from Peprotech, Rocky Hill, NJ) including brain-derived neurotrophic factor (BDNF), glial-derived neurotrophic factor (GDNF), and insulin-like growth factor-1 (IGF1) were added to the cultures of all the groups. To test the requirement of FGF signaling, we treated some of the cells with 5 µM SU5402, a chemical inhibitor of FGF receptors, added at days 4, 6 and 8 of differentiation. The cells were harvested at day 10 of differentiation and processed for fluorescence-activated cell sorting (FACS) analyses as described before[21]. Data were expressed as mean ± standard deviation. The statistical significance for comparison of the SU5402 treated groups with the control groups was analyzed by using Dunnett's test.

### Immunocytochemistry and Quantification

Cells were fixed with 4% paraformaldehyde for 15 min, and incubated in PBS containing 0.2% Triton X-100 (for permeablization) and 10% donkey serum (for blocking). PBS containing 0.1% Triton X-100 and 5% donkey serum was used to dilute the primary antibodies. The cells were incubated with the primary antibodies at 4°C overnight, followed by washing with PBS for three times. Afterwards, the cells were incubated with fluorochrome-conjugated, corresponding secondary antibodies at room temperature for 30 min and washed with PBS-T for three times. Finally, the cells were examined under fluorescence microscope to capture both phase and fluorescent images.

The populations of cells immunostained positive for specific markers among total differentiated cells (all the cell nuclei were counterstained with Hoechst) were counted as described before[16]. In brief, a Zeiss Axio Observer fluorescence microscope (Carl Zeiss Inc., Thornwood, NY) was used to capture images. Then at least 5 fields on each coverslip were randomly chosen and counted [using an ImageJ software program (National Institute of Mental Health, Bethesda, MD)] by an observer blinded to the experimental conditions. Three to four coverslips in each group were counted. Data were expressed as mean ± standard deviation.

### Fluorescence-Activated Cell Sorting Analysis

Cell clumps were harvested by using Accutase (Innovative Cell Technologies Inc., San Diego, CA), and gently dissociated into single cells. The cells were washed with a FACS buffer, which contained PBS, 0.1% NaN_3_, and 2% donkey serum. After being fixed and permeablized with ice-cold 0.1% paraformaldehyde for 10 min and 90% methanol for 30 min, the cells were incubated overnight with the anti-PAX6 antibody or a normal mouse IgG as a negative control at a concentration of one µg of the antibody or IgG per 10^6^ cells. The cells were then washed and incubated with Alexa 488-conjugated donkey anti-mouse IgG for one hour followed by three washing steps. The cells were analyzed on a Becton Dickinson FACS Calibur instrument, and the ratio of PAX6^+^ cells was calculated by using the CellQuest Pro software (BD Biosciences, San Diego, CA).

### Low-density Array Analysis

hESC and hiPSC undergoing neural induction at various time points were collected and subjected to RNA isolation and reverse transcription by using a High Capacity cDNA Reverse Transcription Kit (Applied Biosystems, Foster City, CA), according to the manufacturer's protocol. cDNA derived from approximately 100 ng RNA per sample was applied to TaqMan© Human Stem Cell Pluripotency Low-Density Array card for real-time PCR on an ABI 7900HT Fast System. The samples were tested in triplicate and the data analyzed with RQ2.1 software and displayed as ΔCt (inversely related to mRNA level) in a scatter plot. All the array cards, real-time PCR system, and software were from Applied Biosystems.

### RT-PCR Analysis

RNA was isolated from cells by using TRIzol reagent (Invitrogen), and cDNA was synthesized from the RNA by using ThermoScript (Invitrogen), according to the manufacturers' instructions. Gene expression was assessed through PCR with primers for specific genes (Table S1) under the following conditions for a linear phase of amplification: an initial 5 min denaturation at 95°C; followed by 30 cycles of 45 sec of denaturation at 95°C, 45 sec of annealing at 55°C, and 45 sec of extension at 72°C; and completed with a final extension at 72°C for 10 min.

### Electrophysiology

Coverslips were put in a bath solution including the following (in mM): 1.9 KCl, 26 NaHCO_3_, 2.2 CaCl_2_, 127 NaCl, 1.2 KH_2_PO_4_, 1.4 MgSO_4_, 10 glucose and 10 Hepes at 305 mOsm. Tetrodotoxin (TTX) (1 µM), 4-aminopyridine (4-AP) (1 mM) and tetraethylammonium (TEA) (500 µM) were applied to the cells based on the experimental purpose. Accurate application of drugs was attained using a gravity-fed drug barrel system and all reagents were diluted in extracellular solution. Recording pipettes with resistances of 2–4 MΩ were filled with an intracellular recording solution including the following (in mM): 10 Na^+^-HEPES, 140 K-gluconate, 10 BAPTA, and 4 Mg^2+^-ATP, pH 7.2, 290 mOsm. All chemicals were purchased from Sigma. Neurons were visualized using a Nikon Optical TE2000 microscope (Tokyo, Japan) with differential interference contrast optics at 40×. Voltage-clamp and current-clamp recordings were obtained using an Axopatch 200B amplifier (Molecular Devices, Sunnyvale, CA). Signals were filtered at 4 kHz and sampled at 100 kHz using a Digidata 1322A analog-to-digital converter (Molecular Devices). All data were saved on a computer hard disk and analyzed with pClamp 9.0 (Molecular Devices). Capacitance and series resistance were compensated (typically 50%–80%). The liquid junction potential (∼10 mV) calculated based on previously published methods[36] (JPCalc in Clampex; Molecular Devices) was not adjusted. All recordings were conducted at 21–23°C.

Action potential (AP) amplitude was measured from the threshold to the peak of the voltage deflection. Na^+^ and K^+^ currents were elicited by depolarizing to various voltages from a holding potential of −100 mV. Peak Na^+^ currents and peak K^+^ currents were measured using pClamp 9.0 (Molecular Devices). Transient K^+^ current amplitudes were obtained by measuring the difference between the peak and sustained current amplitude. Na^+^ currents were evaluated by using TTX-sensitive currents.

## Results

### hiPSC Generated by Using Different Systems Form Neural Tube-like Rosettes in Teratomas

The hiPSC colonies started to appear among the fibroblast transduced with the 4 or 6 reprogramming factors at 14–16 days post-transduction. They were isolated and passaged onto new MEF feeder cells, and cultured routinely [30]. Three resultant hiPSC lines, TZ1, YZ1, and YK26, and the hFIB2 hiPSC line from the Daley laboratory [35] were used in this study. TZ1, YZ1, and YK26 were all validated by immunostaining of pluripotency markers and teratoma formation, and all the 4 or 6 transgenes were found silenced in the established hiPSC lines (representative data were shown in Figure S1), The DNA fingerprints of the hiPSC lines all matched their corresponding parental fibroblast lines and the karotypes of the hiPSC lines were all found normal by G-banding (data not shown). For teratoma formation, hiPSC (∼10^7^ cells) grown on MEF feeder cells were harvested and injected into the rear leg muscles of 4-week-old male SCID-beige mice (3 mice per cell line). The mice were sacrificed around 6 to 8 weeks after injection and teratomas excised and examined histologically. Among other germ layer structures, neural tube-like rosettes were found within the ectodermal tissues in the teratomas formed by the hiPSC lines (Figure S1B), which indicates that, like hESC, the hiPSC lines we generated also possess similar ability to form primary neural structures *in vivo*.

### Neural Differentiation Efficiency Differs Among Various hiPSC Lines

**Figure 1:**
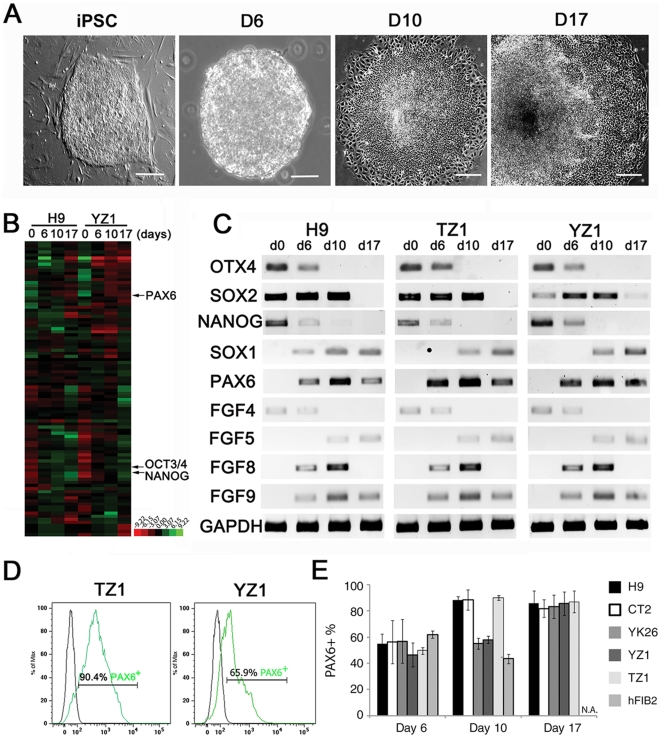
Comparison of neural induction from hiPSC and hESC. (**A**) Morphological changes during neural differentiation from hiPSC. Left to right panels are a hiPSC colony (iPSC - referred to as day 0 of differentiation hereafter), a day-6 EB (d6), day-10 primitive NE cells (d10), and day-17 definitive NE cells (d17). (**B**) Low-density array for gene expression profile in H9 hESC and TZ1 hiPSC during their neural differentiation. Left to right lanes are day 0, 6, 10 and 17 samples as described in **A**. Green color refers to low gene expression (high ΔCt value) and red to high gene expression (low ΔCt value). (**C**) RT-PCR confirmation of the expression patterns of some pluripotent genes and neural differentiation genes. (**D**) Representative histograms for FACS analysis for ratio of PAX6^+^ cells differentiated from TZ1 and YZ1 at day 10. (**E**) Bar chart for FACS analysis for ratio of PAX6^+^ cells at three time points of neural differentiation from two hESC lines and four hiPSC lines. Data from multiple biological replicates are presented as mean ± standard deviation. N.A. stands for not available.

*In vitro* differentiation of hESC to the neural lineage recapitulates the *in vivo* development in multiple aspects, including morphology (formation of neural rosette), timing, and gene expression. Based on our previous experiences with hESC[3], [16], [37], the process of neuroectodermal differentiation starts when hESC detach and aggregate to form embryoid bodies (EBs). After 4-day suspension culture of hiPSC clumps in hESC medium, the hiPSC aggregates were cultured in the neural medium for 2 days and were then plated on a plastic surface. Neural differentiation in the adherent colony culture was examined daily. The attached cells formed individual colonies of monolayer cells 1–2 days later, with increased cell density and compaction in the center of the colonies. After around 10 days in total of differentiation from hiPSC, the cells began to elongate and line up radially to form distinct columns of cells, which were morphologically distinct from the peripheral flat cells that outlined the clusters of columnar cells (primitive NE cells). Continued differentiation for an additional 4–5 days (totally 14–16 days) resulted in the further compaction of the cells and formation of defined ridges of columnar cells. These ridges of columnar cells often formed rings with a distinct inner lumen, a structure reminiscent of the neural tube. Thus, these cellular structures were referred to as “neural tube-like rosettes” (or definite NE, Figure 1A). The morphological changes during neural differentiation were very similar between hESC and hiPSC.

We then analyzed the gene expression profiles using a low-density array, which are shown in a heatmap (Figure 1B) with the raw data presented Table S2. Through RT-PCR (Figure 1C), we confirmed that expression of the pluripotency genes *POU5F1* (*OCT4*) and *NANOG* decreased starting at day 6 of differentiation from either hESC (H9) or hiPSC (YZ1). In contrast, *SOX2*, expressed by both hESC/hiPSC and neural stem cells, was highly expressed in H9, YZ1, and early neural cells differentiated from the two cell lines. Meanwhile, expression of neural specific makers, e.g., *PAX6* and *SOX1*, increased during differentiation (Figures 1C).

To compare the efficiency of neural differentiation between the different cell lines, we analyzed the ratios of PAX6^+^ NE cells from two hESC lines H9 and CT2 and four hiPSC lines YK26, YZ1, TZ1, and hFIB2 by FACS at multiple time points (Figures 1D and 1E). As a general neural stem cell marker and early neural transcription factor, PAX6 protein is detectable as early as day 6 of neural differentiation from hESC [16], [37]. TZ1 matched the hESC lines very well in neural differentiation efficiency. However, YK26 and YZ1 differentiated slower than TZ1, H9, and CT2, as their PAX6+ cell ratios lagged behind at day 10 but caught up at day 17. The fourth hiPSC line hFIB2 behaved even more differently than the others, the hFIB2 cells attached poorly and detached easily resulting in a decline in PAX6+ cell ratio at day 10 and no cells available by day 17. These results suggest that heterogeneity indeed exist among various hiPSC lines, and timing of differentiation is also a matter.

### FGF Signaling is Required for Early Neural Induction

**Figure 2:**
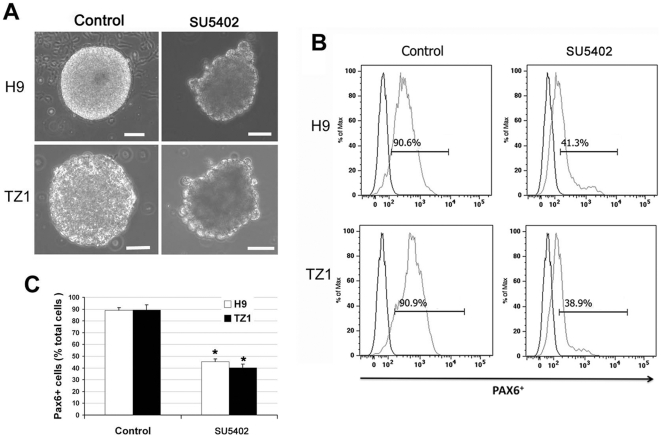
Requirement of FGF signaling for neural induction from hiPSC and hESC. (**A**) Phase contrast images for EBs at day 8 of neural differentiation from H9 hESC and TZ1 hiPSC treated with 5 µM SU5402 or vehicle (Control) from days 4 through 8. (**B**) Decline of PAX6^+^ cell ratio detected by FACS at day 10 of neural differentiation from H9 and TZ1 cells treated with SU5402 or vehicle on the last 6 days. (**C**) The decline of PAX6^+^ cell ratio from **B** was analyzed statistically. Data are presented as mean ± standard deviation. n = 4. *P<0.05 versus the control group.

By using the *Xenopus* embryo, we have previously demonstrated that inhibition of the BMP pathway is sufficient for neural induction[8] and activation of FGF pathway is required for both neural induction and caudalization[38]. Other studies have indicated the involvement of these pathways in neural differentiation from mouse[39], [40] and human[3], [16], [41] ES cells. We noticed that expression of some FGF members (e.g., *FGF8* and *FGF9*) was up-regulated during early neural induction from hiPSC as well hESC (Figure 1C), which led us to evaluate the role of the FGF pathway during neural induction from hiPSC. We blocked FGF signaling at the receptor level by using SU5402[42]. We treated TZ1 hiPSC or H9 hESC with 5 µM SU5402 from days 4 through 8, and found that there were no clear morphological differences in the beginning of the EB formation between the treated and control cells (data not shown). The ratio of PAX6^+^ cells differentiated from TZ1 dramatically declined at day 10 of differentiation compared to that of the control groups (Figures 2B and 2C). These data suggest that FGF signaling is required for neural induction from hiPSC as well as hESC.

### Region-specific Neural Differentiation from hiPSC

**Figure 3:**
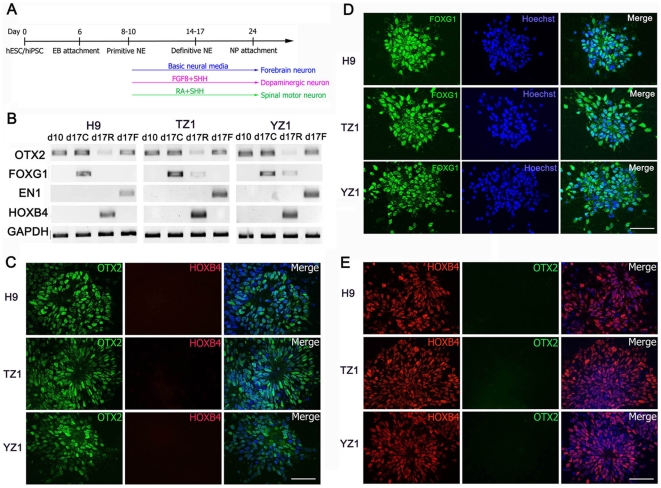
Differentiation of hiPSC/hESC-derived NE cells into region-specific neural progenitors. (**A**) Schematic for protocols to generate region-specific neural progenitors. (**B**) RT-PCR analysis for expression of anterior-posterior neural marker genes at days 10 (d10) and 17 (d17) of neural differentiation from H9 hESC and TZ1 and YZ1 hiPSC lines. The day-17 cells were treated with RA (d17R) or FGF8 (d17F) for the last 7 days with untreated cells as a control (d17C). (**C & D**) Immunostaining for OTX2 and HOXB4 (**C**) or FOXG1 (**D**) on neural progenitors differentiated for 25 days from H9, TZ1, and YZ1. Cell nuclei were counterstained with Hoechst 33342. Bar, 50 µm. (**E**) The same staining on neural progenitors differentiated for 25 days from the 3 cell lines that were treated with RA.

The protocols we used for generation of region-specific neural cells from hiPSC were similar to those developed for hESC[16], [18], [21] (Figure 1A). In the absence of exogenous growth factors, NE cells differentiated from either hESC or hiPSC expressed the anterior transcription factor *OTX2*, which was detected at days 10 and 17 of differentiation (Figure 3B). Expression of the telencephalic transcription factor *FOXG1* ^28^ was detected in the NE cells by RT-PCR at day 17 of differentiation (Figure 3B) and by immunostaining at day 25 of differentiation (Figure 3D). Immunostaining at day 25 and counting of the stained cells (see Materials and Methods) demonstrated that OTX2^+^ cells were approximately 87.8±5.81%, 85.5±6.80%, and 82.3±7.09% among the NE cells differentiated from H9, TZ1, and YZ1 groups, respectively, whereas the hindbrain marker HOXB4 was absent in all the NE cells. The predominant and persistent expression of the anterior markers was accompanied by lack of expression of *EN1* and *HOXB4*, two transcriptional factors expressed in the mid/hind-brain and spinal cord cells, as assayed by RT-PCR (Figure 3B).

To test whether these forebrain neural progenitors could be caudalized by addition of morphogens, we added 50 ng/ml FGF8 (for induction of the midbrain cells) or 0.1 µM RA (for induction of the midbrain and hindbrain cells) to the culture of the NE cells starting at day 10 of differentiation. Treatment with FGF8 induced expression of the midbrain marker *EN1* at day 17 (the d17F lane in Figure 3B) in the NE cells differentiated from both hiPSC and hESC groups. In contrast, treatment with RA induced NE cells from both groups to express *HOXB4*, a marker for the hindbrain and spinal cord (the d17R lane in Figures 3B and 3E). At day 25, 92.7±5.1%, 93.2±4.5%, and 91.6±5.1% in the H9, TZ1, YZ1 groups, respectively, were positive for HOXB4 and all were negative for OTX2 (Figure 3E). This is in sharp contrast to the control NE cells induced in the absence of the morphogens (the d17C lane in Figures 3B and 3C). Together, these data suggest that hiPSC-derived NE cells can be efficiently caudalized along the anterior-posterior axis following treatment with these morphogens.

### Differentiation of Functional Neurons from Region-specific Progenitors

**Figure 4:**
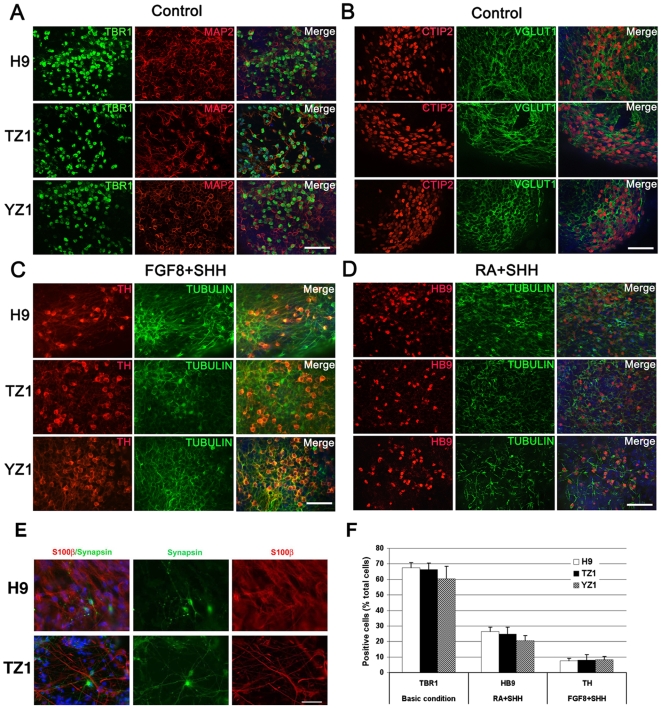
Further differentiation of hiPSC/hESC-derived neural progenitors into region-specific neurons and astrocytes. (**A & B**) Immunostaining for the forebrain functional markers TBR1 and MAP2 (**A**), and CTIP2 and VGLUT1 (**B**) on cells differentiated for 5 (**A**) or 6 (**B**) weeks from H9 hESC and TZ1 and YZ1 hiPSC lines. (**C**) Immunostaining for the dopaminergic neuron marker TH on cells differentiated from hESC/hiPSC-derived and FGF8/SHH-treated neural progenitors. (**D**) Immunostaining for the spinal motor neuronal marker HB9 (with βIII-tubulin as a neuronal control marker) on cells differentiated from hESC/hiPSC-derived and RA/SHH-treated neural progenitors. (**E**) Some cells were positive for S100β (an astrocyte marker) or Synapsin at two months after differentiation from H9 or TZ1 cells. Cell nuclei were counterstained with Hoechst 33342. Bar, 50 µm. (**F**) Percentage of cells immunostained positive for TBR1, HB9, and TH counted for **A**, **C**, and **D**, respectively.

Among the most common neurotransmitters in the brain, glutamate mainly initiates excitatory signals and GABA initiates inhibitory signals. Differentiation into glutamatergic and GABAergic neurons indicates the maturation of the forebrain progenitors. To test this maturation, we plated hESC/hiPSC-derived forebrain progenitor cells on coverslips for differentiation in the absence of morphogens for 5 weeks. At this time point, a large population (>60%) of cells expressed *TBR1* (Figures 4A and 4F), a transcriptional factor expressed by glutamatergic neurons, and many TBR1^+^ neurons also expressed microtubule-associated protein 2 (MAP2), a mature neuron marker (Figure 4A). Some neurons were positive for CTIP2 (Figure 4B), a transcriptional factor expressed by subcerebral projection neurons. Moreover, almost all the CTIP2^+^ cells were also positive for vesicular glutamate transporter 1 (VGLUT1) (Figure 4B), a marker expressed by mature glutamatergic neurons[39]. These results indicate that the forebrain progenitors derived from hiPSC, similar to those from hESC, can further differentiate into forebrain glutamatergic neurons following maturation from NE cells to dorsal telencephalic cells in absence of known morphogens.

Previously, we [21] and others [43] have shown that the above treatments not only generate GABAergic neurons but also various types of glia. Here we also found that S100β^+^ astrocytes were present among the cells differentiated for two months from both H9 hESC and TZ1 hiPSC (Figure 4E). Synapsin^+^ neurons were also identified among both the H9- and TZ1-differentiated cells, suggesting that these neurons can make synapses in the long-term culture (Figure 4E). Percentages of cells immunostained positive for TBR1 counted in the above assays were similar between the hESC and hiPSC lines (Figure 4F).

In order to generate dopaminergic neurons and spinal motor neurons, the NE-derived midbrain (induced by FGF8) and spinal progenitors (induced by RA) were ventralized by treatment with 100 ng/ml SHH (R&D Systems) at days 10–17 and days 17–31, respectively. After suspension culture in the neural medium for one week from days 17 through 24, these neural progenitors were plated on poly-ornithine/laminin coated coverslips for terminal differentiation. Following another 2-week differentiation (totally 5 weeks), dopaminergic (TH^+^) and spinal motor (HB9^+^) neurons were generated from the FGF8/SHH- and RA/SHH-treated NE cells, respectively (Figures 4C and 4D). Counting of the positively immunostained neurons showed comparable ratios of the TH^+^ (<10%) and HB9^+^ (∼20%) neurons among differentiated cells from H9, TZ1, and YZ1 cell lines (Figure 4F).

**Figure 5:**
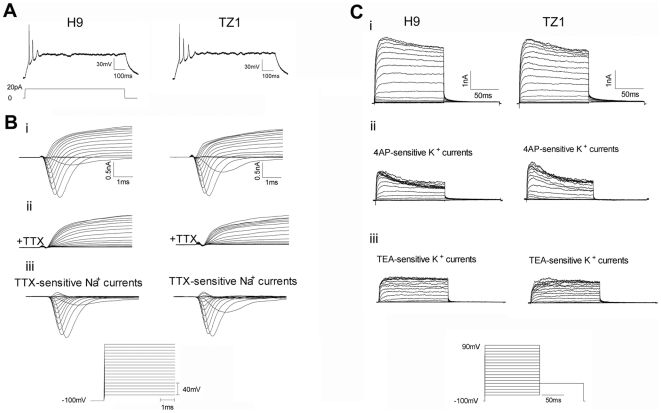
hiPSC-derived neurons are functional ***in vitro***. (**A**) Action potentials (APs) were observed, representative voltage responses to a 20 pA current injection are shown for neurons following 6 weeks of differentiation from H9 and TZ1 cells in the basic neural induction condition without exogenous morphogens. (**B**) (**i**) Rapidly activating and inactivating voltage-gated inward currents were elicited by depolarizing to various voltages from a holding potential of −100 mV. (**ii**) The inward currents were completely blocked by TTX (1 µM). (**iii**) TTX-sensitive Na^+^ current in H9 and TZ1 cells. (**C**) (**i**) Representative traces showing fast inactivating and sustained-outward currents elicited by voltage steps from a holding potential of −100 mV. 4AP (1 mM) eliminated the fast inactivating K^+^ current, and TEA (0.5 mM) blocked the sustained currents. (**ii**) 4AP-sensitive K^+^ currents. (**iii**) TEA-sensitive K^+^ currents. Values of the electrophysiological parameters detected in representative neurons differentiated from both H9 and TZ1 cells are shown in Table S3.

To determine the function of the hiPSC-derived neurons, we examined the electrophysiological properties of neurons at 6–8 weeks of differentiation from TZ1 hiPSC, in comparison to those from H9 hESC. Neurons differentiated from both H9 and TZ1 cells in the basic condition without exogenous morphogens had a similar ability to fire action potentials (APs) in response to depolarizing current pulses (Figure 5A). Notably, both Na^+^ currents and K^+^ currents contribute to the APs detected from both groups of neurons. Large and rapidly activating inward currents were reliably induced by voltage steps from a holding potential of −100 mV (Figure 5Bi) and were blocked completely by TTX applied to the extracellular solution (Figure 5Bii).

To determine whether outward K^+^ currents contributed to the APs, we analyzed outward currents in response to voltage steps from a holding potential of −100 mV (Figure 5Ci). We observed two distinct K^+^ current components. The transient outward current could be isolated by subtracting the 4-AP-treated current (Figure 5Cii) from the untreated current, the remaining sustained current was present in all cells and could be reduced with 0.5 mM TEA (Figure 5Ciii). Similar results were obtained between recordings of the above electrophysiological parameters on 30 H9-derived and 40 TZ1-derived neurons (Table S3). These results suggest that differentiating neuronal cells from hESC and hiPSC are functionally alike to each other, as both can fire APs, and gain characteristic Na^+^ and K^+^ currents at 6–8 weeks of differentiation. The ability of the neurons to fire APs appears age-dependent because we did not detect APs and Na+/K+ currents in younger neurons (data not shown).

## Discussion

A paucity of reliable neural disease models has been a major hurdle for studying pathologic mechanisms, screening new drugs, and developing new therapies to treat neural degenerative diseases. Similar to hESC, hiPSC derived from somatic cells possess self-renewal and pluripotency properties and are expected to serve as a powerful tool to model diseases for basic and therapeutic research[22], [23], [26], [27], [28]. Although great efforts have been made to explore the similarities and differences between hESC and hiPSC at the pluripotent stage[44], little is known about whether these two cell types have similar abilities to differentiate into functional cells of specific lineages. In this paper, we have demonstrated differentiation of hiPSC into a spectrum of region-specific neural progenitors, which further develop into functional neurons. We have also revealed the heterogeneity among various hiPSC lines to undergo neural differentiation.

Neural induction is the first step during neural development[45], [46]. hESC differentiation into neural cells can be triggered and enhanced *in vitro* by using morphogens such as BMP antagonists and FGF agonists that are also critical for *in vivo* development of the neural ectoderm[3], [47], [48]. By applying a BMP antagonist alone or together with SB431542 (a small molecule inhibitor of the Nodal/Activin/TGFβ pathway), both hESC and hiPSC can be efficiently differentiated into NE cells[4]. Another commonly used method for neural induction is via EB formation in a chemically defined system including a minimum medium, which mimics the development of neural ectoderm cells in morphology and gene expression profiles[3], [5], [37]. Although addition of bFGF into this system may increase the number of NE cells, NE cells can be efficiently generated from hESC without bFGF. Further analysis has shown that endogenous FGF contributes to this process, irrespective of the inhibition of BMP signaling[49]. In our present study, we used a chemically defined system and carefully analyzed the initial and terminal neural differentiation of various hiPSC lines in comparison to the H9 hESC line. We found that the morphological changes and gene expression patterns during neural differentiation from hiPSC are very similar to those for hESC. Addition of SU5402, the inhibitor of FGF receptors, significantly decreased the generation of PAX6^+^ NE cells, which suggests that FGF signaling is also required for neural induction from hiPSC as well as hESC.

Using FACS analysis to quantify PAX6^+^ NE cells, we compared the neural differentiation efficiency among four hiPSC lines and two hESC lines (Figure 1E). We observed three scenarios in terms of the differentiation efficiency: 1) comparable with hESC (for TZ1); 2) low but eventually catching up (for YK26 and YZ1); and 3) poor due to cell attachment problem (for hFIB2). These variations suggest that heterogeneity indeed exists among various hiPSC lines, which has also been shown in a recent study [43]. The reasons for the heterogeneity are not clear. Recent studies showed quite different gene expression patterns and miRNA expression profiles among various hiPSC lines [44], [50]. Even the gene expression pattern for late-passage hiPSC is different from that for early-passage hiPSC[44]. Our low-density array data also showed marked variations between the YZ1 hiPSC line and H9 hESC line in gene expression profile during their neural differentiation. Whether the variations in neural differentiation efficiency are due to differences in pluripotency levels or other intrinsic nature of the cell lines awaits further investigation. Our results also argue for the importance to use multiple hiPSC lines and test at multiple time points before a conclusion is drawn as both cell line heterogeneity and differentiation timing are impact factors.

Although differentiation of dopaminergic neurons and motor neurons from hiPSC has been reported[4], [51], the ability of hiPSC/hESC-derived NE cells to further differentiate into various region-specific progenitors is largely unknown. In this paper, we induced hiPSC differentiation into a spectrum of region-specific neural cells by using various morphogens and compared the efficiency of such differentiation between various hiPSC lines. Although we observed different efficiencies of neural induction from various hiPSC lines, NE cells derived from both TZ1 and YZ1 could further differentiate into forebrain, midbrain, and spinal cord progenitors. The generation of the region-specific neural cells positive for FOXG1, OTX2 or HOXB4 was comparable between TZ1 and YZ1 hiPSC, and H9 hESC. These neural progenitors could further differentiate into functional glutamatergic, dopaminergic, and spinal motor neurons as well as astrocytes by using protocols developed for hESC[16], [18], [21]. Together, our work has demonstrated that hiPSC, regardless how they are derived, can generate region-specific neurons including the forebrain glutamatergic neurons.

## Supporting Information

Figure S1 Characterization of hiPSC. (A) Immunostaining for pluripotency markers SSEA4 and TRA-1-60 on H9 hESC and TZ1 and YZ1 hiPSC lines. (B) Teratomas formed at 6-8 weeks after TZ1, YZ1, and YK26 cells were injected intramuscularly into NOD/SCID mice. Representative tissues from the three germ layers are shown. (C) RT-PCR analysis for expression of the reprogramming/pluripotency genes in H9, TZ1, and YZ1 cells. Primers that recognized both the endogenous (Endo) and total (Total) genes (including the transduced genes) were used to detect the silencing of the transduced genes.
(7.91 MB TIF)

Table S1 (0.04 MB DOC)

Table S2 (0.16 MB DOC)

Table S3 (0.03 MB DOC)
