# Supplementary figures and images for: Specification of Region-Specific Neurons Including Forebrain Glutamatergic Neurons from Human Induced Pluripotent Stem Cells
Source: PLoS One. 2010 Jul 29;5(7):e11853. doi: 10.1371/journal.pone.0011853 (PMC2912324; doi:10.1371/journal.pone.0011853)

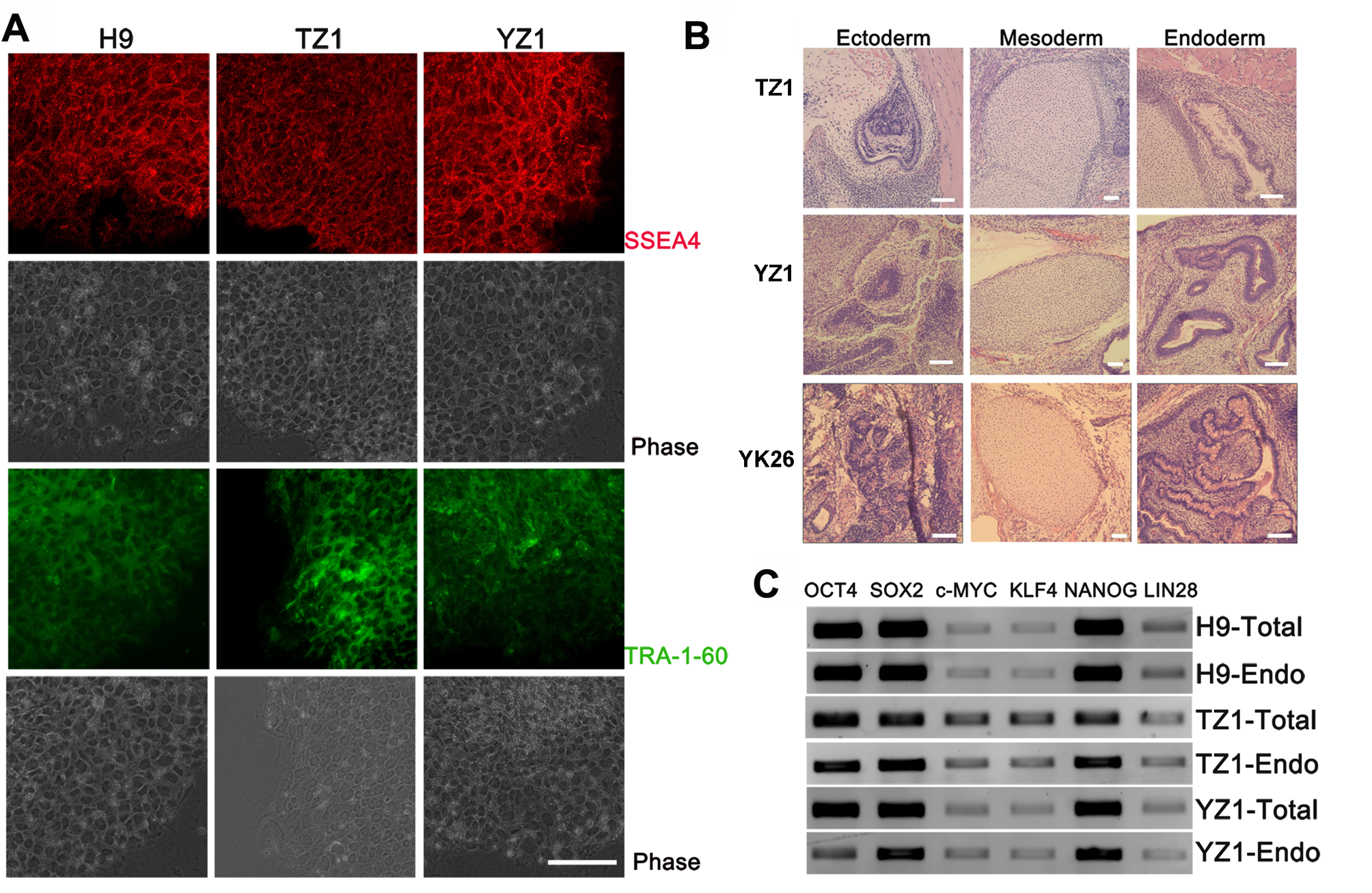

Supplement: Figure S1 — Characterization of hiPSC. (A) Immunostaining for pluripotency markers SSEA4 and TRA-1-60 on H9 hESC and TZ1 and YZ1 hiPSC lines. (B) Teratomas formed at 6-8 weeks after TZ1, YZ1, and YK26 cells were injected intramuscularly into NOD/SCID mice. Representative tissues from the three germ layers are shown. (C) RT-PCR analysis for expression of the reprogramming/pluripotency genes in H9, TZ1, and YZ1 cells. Primers that recognized both the endogenous (Endo) and total (Total) genes (including the transduced genes) were used to detect the silencing of the transduced genes. (7.91 MB TIF) [file pone.0011853.s001.tif]
