# Supplementary material for: Specification of Region-Specific Neurons Including Forebrain Glutamatergic Neurons from Human Induced Pluripotent Stem Cells
Source: PLoS One. 2010 Jul 29;5(7):e11853. doi: 10.1371/journal.pone.0011853 (PMC2912324; doi:10.1371/journal.pone.0011853)
Supplement: Table S1 — (0.04 MB DOC) [file pone.0011853.s002.doc]

**Table S1. Primers for RT-PCR**

| **Gene** | **Forward** | **Reverse** |
| --- | --- | --- |
| OCT4-Total | 5’-CAGTGCCCGAAACCCACAC | 5’-GGAGACCCAGCAGCCTCAAA |
| OCT4-Endo | 5’-AGTTTGTGCCAGGGTTTTTG | 5’-ACTTCACCTTCCCTCCAACC |
| SOX2-Total | 5’-TACCTCTTCCTCCCACTCCA | 5’-GGTAGTGCTGGGACATGTGA |
| SOX2-Endo | 5’-AGTCTCCAAGCGACGAAAAA | 5’-TTTCACGTTTGCAACTGTCC |
| c-MYC-Total | 5’-ACTCTGAGGAGGAACAAGAA | 5’-TGGAGACGTGGCACCTCTT |
| c-MYC-Endo | 5’-TGCCTCAAATTGGACTTTGG | 5’-GATTGAAATTCTGTGTAACTGC |
| KLF4-Total | 5’-TCTCAAGGCACACCTGCGAA | 5’-TAGTGCCTGGTCAGTTCATC |
| KLF4-Endo | 5’-GATGAACTGACCAGGCACTA | 5’-GTGGGTCATATCCACTGTCT |
| NANOG-Total | 5’-TTTGGAAGCTGCTGGGGAAG | 5’-GATGGGAGGAGGGGAGAGGA |
| NANOG-Endo | 5’-CAGAAGGCCTCAGCACCTAC | 5’-ATTGTTCCAGGTCTGGTTGC |
| LIN28-Total | 5’-AAGCGCAGATCAAAAGGAGA | 5’-CTGATGCTCTGGCAGAAGTG |
| LIN28-Endo | 5’-AGTGGCCTGGATAGGGAAGT | 5’-CTTGGCTCCATGAATCTGGT |
| OTX2 | 5’-GACCCGGTACCCAGACATC | 5’-TGGCCACTTGTTCCACTCTC |
| FOXG1 | 5’-AGAAGAACGGCAAGTACGAGA | 5’-TGTTGAGGGACAGATTGTGGC |
| EN1 | 5’-GGACAATGACGTTGAAACGCAGCA | 5’-AAGGTCGTAAGCGGTTTGGCTAGA |
| HOXB4 | 5’-AAAAGAGCCCGTCGTCTACC | 5’-GTGTAGGCGGTCCGAGAG |
