# Supplementary material for: Specification of Region-Specific Neurons Including Forebrain Glutamatergic Neurons from Human Induced Pluripotent Stem Cells
Source: PLoS One. 2010 Jul 29;5(7):e11853. doi: 10.1371/journal.pone.0011853 (PMC2912324; doi:10.1371/journal.pone.0011853)
Supplement: Table S2 — (0.16 MB DOC) [file pone.0011853.s003.doc]

**Table S2. Raw data for low-density array to compare gene expression during neural differentiation from H9 hESC and YZ1 hiPSC**

| **Detector** | **H9 D0** | **H9 D6** | **H9 D10** | **H9 D17** | **YZ1 D0** | **YZ1 D6** | **YZ1 D10** | **YZ1 D17** |
| --- | --- | --- | --- | --- | --- | --- | --- | --- |
| ACTC-Hs00606316_m1 | 0.90 | 2.54 | -0.54 | -1.13 | 1.32 | -0.36 | -2.92 | 0.20 |
| FN1-Hs00277509_m1 | -0.61 | 2.73 | -0.23 | -0.52 | 1.63 | 0.05 | -2.04 | -1.01 |
| GATA6-Hs00232018_m1 | 0.50 | 1.38 | 0.81 | 0.22 | 1.16 | 0.13 | -2.80 | -1.40 |
| COL1A1-Hs00164004_m1 | -0.08 | 3.56 | 0.47 | -1.94 | 1.68 | 1.06 | -1.48 | -3.27 |
| IL6ST-Hs00174360_m1 | 1.20 | 1.65 | -0.02 | -1.62 | 1.99 | 0.18 | -1.53 | -1.84 |
| AFP-Hs00173490_m1 | 0.16 | 11.06 | 1.01 | -4.65 | 9.53 | -6.74 | -4.08 | -6.29 |
| SERPINA1-Hs00165475_m1 | 2.17 | 3.06 | 2.72 | -2.54 | 3.62 | -3.12 | -1.57 | -4.35 |
| HBZ-Hs00744391_s1 | 1.14 | 6.75 | 2.13 | -0.78 | 1.19 | -4.91 | -3.26 | -2.27 |
| SST-Hs00174949_m1 | 0.77 | 2.35 | 2.32 | 0.73 | 0.82 | -2.20 | -2.54 | -2.27 |
| CD34-Hs00156373_m1 | -2.06 | 0.89 | 1.84 | -1.56 | 4.44 | 0.26 | -2.22 | -1.58 |
| PTEN-Hs00829813_s1 | -1.89 | -0.12 | 0.76 | 0.07 | 4.48 | 0.94 | -3.04 | -1.20 |
| RUNX2-Hs00231692_m1 | 0.76 | 0.25 | -0.55 | -2.46 | 5.66 | 0.14 | -2.16 | -1.63 |
| LIFR-Hs00158730_m1 | -0.99 | 0.14 | -0.04 | 1.44 | 4.60 | -3.00 | -1.62 | -0.54 |
| CDH5-Hs00174344_m1 | 4.96 | -0.61 | -1.12 | -0.75 | 1.97 | 1.06 | -2.77 | -2.74 |
| COL2A1-Hs00156568_m1 | 3.48 | 0.53 | -0.94 | -2.20 | 2.31 | 0.54 | -2.40 | -1.33 |
| NOG-Hs00271352_s1 | 2.12 | -0.18 | -0.31 | -0.75 | 1.67 | 0.85 | -2.58 | -0.82 |
| NEUROD1-Hs00159598_m1 | 4.51 | 0.80 | -2.00 | -2.27 | 4.56 | -1.04 | -2.51 | -2.07 |
| PAX6-Hs00240871_m1 | 4.14 | -0.43 | -1.75 | -2.88 | 5.80 | 0.06 | -2.35 | -2.58 |
| COMMD3-Hs00201350_m1 | 2.56 | 1.27 | 0.92 | -1.60 | 0.21 | -0.68 | -0.65 | -2.03 |
| PECAM1-Hs00169777_m1 | 1.32 | 0.34 | 1.47 | -1.06 | 1.29 | -0.41 | 0.15 | -3.11 |
| ISL1-Hs00158126_m1 | 3.75 | -0.92 | 0.62 | 0.19 | 2.57 | -3.27 | -1.30 | -1.64 |
| HBB-Hs00747223_g1 | 4.53 | 0.11 | 2.23 | -2.62 | -3.26 | -1.84 | 0.13 | 0.70 |
| EOMES-Hs00172872_m1 | 0.36 | 1.82 | 1.24 | -0.89 | -0.32 | -1.07 | -1.66 | 0.51 |
| FGF5-Hs00170454_m1 | 1.16 | 2.74 | 2.71 | -4.74 | 1.21 | -1.25 | -2.95 | 1.12 |
| TH-Hs00165941_m1 | 1.02 | 2.60 | 2.57 | -4.14 | -2.99 | 1.61 | -1.65 | 0.98 |
| GATA4-Hs00171403_m1 | -1.18 | 1.90 | 1.62 | 0.49 | 0.06 | -0.52 | -1.34 | -1.02 |
| FOXA2-Hs00232764_m1 | -1.81 | 1.36 | 1.00 | 0.69 | -0.47 | -0.57 | -1.02 | 0.82 |
| SOX17-Hs00751752_s1 | -1.35 | 2.47 | 1.57 | 1.42 | 0.04 | -2.11 | -2.52 | 0.49 |
| CDX2-Hs00230919_m1 | 0.93 | 1.21 | 6.57 | -0.24 | 1.00 | -3.23 | -3.92 | -2.31 |
| Xist-Hs01079824_m1 | 1.95 | -0.62 | 3.50 | 1.91 | 2.00 | -5.56 | -5.09 | 1.91 |
| CRABP2-Hs00275636_m1 | 0.89 | -0.70 | -1.17 | 0.04 | 1.37 | -0.19 | -1.19 | 0.96 |
| GCM1-Hs00172692_m1 | 1.54 | -1.45 | -0.97 | 1.50 | 1.59 | -1.29 | -2.42 | 1.50 |
| ACTB-Hs99999903_m1 | -0.32 | -0.32 | -1.01 | 0.81 | -0.62 | 1.16 | -1.66 | 1.95 |
| IMP2-Hs00538956_m1 | 0.28 | -0.38 | -0.82 | 0.40 | -0.17 | 0.50 | -0.84 | 1.03 |
| GBX2-Hs00230965_m1 | 0.34 | -0.96 | -1.35 | 1.03 | -0.01 | 0.21 | -0.24 | 0.98 |
| HLXB9-Hs00232128_m1 | 1.15 | -1.73 | 2.70 | 1.11 | -2.81 | 1.74 | -3.27 | 1.11 |
| CGB-Hs00361224_gH | 2.68 | 1.03 | -0.83 | -0.35 | -0.91 | 0.60 | -1.77 | -0.45 |
| T-Hs00610080_m1 | 3.67 | -0.07 | -1.43 | 3.63 | -0.30 | -1.55 | -4.30 | 0.34 |
| CTNNB1-Hs00170025_m1 | 0.00 | 0.00 | 0.00 | 0.00 | 0.00 | 0.00 | 0.00 | 0.00 |
| 18S-Hs99999901_s1 | -1.29 | -0.35 | -0.46 | -0.59 | 0.67 | 0.76 | -1.12 | 2.38 |
| LAMA1-Hs00300550_m1 | -0.65 | -0.18 | -0.74 | -0.26 | 0.78 | 0.66 | -0.55 | 0.96 |
| RAF1-Hs00234119_m1 | -0.59 | -0.18 | -0.37 | -0.23 | -0.17 | 0.89 | -0.32 | 0.97 |
| OLIG2-Hs00377820_m1 | -1.96 | -0.33 | -1.46 | -1.73 | 0.71 | 1.90 | 0.63 | 2.23 |
| SOX2-Hs00602736_s1 | -1.36 | -0.60 | -0.42 | -0.69 | 0.48 | 0.29 | 0.14 | 2.17 |
| NES-Hs00707120_s1 | 0.21 | 0.43 | -1.14 | -0.20 | 0.76 | 0.61 | -1.48 | 0.81 |
| SFRP2-Hs00293258_m1 | 0.57 | 0.44 | -0.88 | -1.31 | 0.83 | 1.21 | -1.74 | 0.87 |
| SEMA3A-Hs00173810_m1 | 0.20 | -0.01 | -0.03 | -0.93 | 0.16 | 0.59 | -0.36 | 0.37 |
| FOXD3-Hs00255287_s1 | -3.61 | -1.47 | 0.15 | 4.17 | -2.84 | 0.75 | -1.32 | 4.17 |
| FGF4-Hs00173564_m1 | -2.26 | -0.68 | -1.65 | 4.08 | -3.03 | -0.33 | -0.32 | 4.20 |
| GFAP-Hs00157674_m1 | -2.70 | -0.21 | -0.54 | 2.74 | -1.22 | -0.87 | 0.06 | 2.74 |
| EEF1A1-Hs00742749_s1 | -1.68 | -0.05 | -0.62 | 1.26 | -0.50 | 0.04 | -0.64 | 2.19 |
| TERT-Hs00162669_m1 | -2.81 | -1.34 | -1.31 | 2.04 | -2.57 | 0.00 | -0.10 | 6.09 |
| LIN28-Hs00702808_s1 | -1.46 | -1.25 | -0.52 | 1.35 | -0.73 | -0.52 | -0.68 | 3.79 |
| NR6A1-Hs00265966_m1 | -0.79 | -1.41 | -0.68 | 1.19 | -0.20 | -0.29 | -0.53 | 2.70 |
| LAMB1-Hs00158620_m1 | -0.48 | 0.10 | -0.10 | 0.31 | -0.20 | 0.25 | -0.55 | 0.66 |
| GAPD-Hs99999905_m1 | 0.13 | 0.20 | 0.25 | 0.47 | -0.84 | -0.27 | -0.93 | 1.00 |
| LAMC1-Hs00267056_m1 | -0.10 | 0.52 | -0.21 | -0.09 | -1.14 | 0.49 | -0.72 | 1.27 |
| REST-Hs00194498_m1 | 0.15 | -0.09 | -0.08 | 0.30 | -0.87 | 0.43 | -0.68 | 0.84 |
| PAX4-Hs00173014_m1 | 1.61 | -1.94 | -3.84 | 1.57 | -4.13 | 2.20 | 2.97 | 1.57 |
| BRIX-Hs00217848_m1 | -0.88 | 0.17 | 0.89 | 0.21 | -0.85 | -0.67 | -0.16 | 1.30 |
| SYCP3-Hs00538143_m1 | -2.23 | 3.75 | 3.72 | -1.31 | -2.70 | -1.83 | -1.53 | 2.13 |
| FLT1-Hs00176573_m1 | -2.04 | -0.14 | 2.59 | 0.81 | -2.38 | 1.03 | 0.34 | -0.21 |
| TFCP2L1-Hs00232708_m1 | -2.20 | -1.04 | 5.55 | 3.97 | -3.31 | -1.80 | -1.16 | -0.02 |
| GDF3-Hs00220998_m1 | -1.67 | 0.38 | 0.80 | 7.29 | -4.97 | -2.14 | -1.88 | 2.17 |
| NODAL-Hs00415443_m1 | -0.72 | 0.93 | 1.17 | 2.88 | -3.75 | -0.61 | -1.93 | 2.03 |
| DNMT3B-Hs00171876_m1 | -2.46 | -1.07 | 0.50 | 2.20 | -2.60 | -0.51 | 0.76 | 3.20 |
| GRB7-Hs00917999_g1 | -2.70 | -0.41 | 0.33 | 1.77 | -1.66 | -0.22 | 0.45 | 2.44 |
| KIT-Hs00174029_m1 | -0.92 | 0.14 | 0.17 | 0.37 | -1.24 | 0.32 | 0.15 | 1.02 |
| CD9-Hs00233521_m1 | -1.60 | 1.15 | 1.85 | 1.31 | -2.71 | 0.11 | -0.48 | 0.38 |
| GAL-Hs00544355_m1 | -2.25 | 0.91 | 2.43 | 1.88 | -4.86 | -0.83 | 0.04 | 2.68 |
| IFITM1-Hs00705137_s1 | -1.79 | 0.17 | 2.35 | 1.56 | -3.38 | -0.76 | 0.81 | 1.04 |
| LEFTB-Hs00764128_s1 | -3.77 | 2.71 | 2.75 | 0.88 | -4.94 | -1.36 | 0.77 | 2.95 |
| NR5A2-Hs00187067_m1 | -2.11 | 0.69 | 1.57 | 0.83 | -2.07 | -0.02 | 0.36 | 0.75 |
| POU5F1-Hs00742896_s1 | -3.71 | 0.91 | 1.23 | 2.12 | -3.31 | -0.14 | 1.47 | 1.43 |
| GABRB3-Hs00241459_m1 | -2.18 | 0.20 | 0.96 | 1.90 | -2.38 | -0.61 | 0.39 | 1.73 |
| Nanog*-Hs02387400_g1 | -3.76 | 0.52 | 1.83 | 4.65 | -4.39 | -1.24 | -0.30 | 2.67 |
| TDGF1-Hs02339499_g1 | -3.68 | 0.34 | 2.41 | 4.69 | -5.14 | -1.66 | -0.11 | 3.15 |
| ZFP42-Hs00399279_m1 | -2.62 | -0.92 | 1.68 | 3.32 | -2.00 | -1.49 | 0.06 | 1.96 |
| PODXL-Hs00193638_m1 | -2.58 | 0.44 | 1.58 | 1.44 | -0.92 | -0.33 | -0.22 | 0.58 |
| MYOD1-Hs00159528_m1 | -0.13 | -2.57 | 1.42 | -0.17 | -0.08 | 0.46 | 1.23 | -0.17 |
| UTF1-Hs00747497_g1 | -4.03 | -2.44 | 2.72 | 1.13 | 1.22 | 1.76 | -1.49 | 1.13 |
| EBAF-Hs00745761_s1 | -2.03 | 3.95 | 1.10 | -2.45 | -2.45 | 0.69 | 0.33 | 0.86 |
| IPF1-Hs00236830_m1 | -0.63 | 0.95 | 0.92 | -0.67 | -0.58 | -0.04 | 0.73 | -0.67 |
| DDX4-Hs00251859_m1 | -0.12 | 1.45 | 1.42 | -4.21 | -0.07 | 0.46 | 1.23 | -0.16 |
| SYP-Hs00300531_m1 | -1.47 | 0.49 | 1.70 | -1.89 | -0.54 | 0.24 | 1.11 | 0.35 |
| IFITM2-Hs00829485_sH | -0.71 | 0.22 | 0.32 | -0.52 | -1.90 | 0.90 | 0.89 | 0.82 |
| MYF5-Hs00271574_m1 | -0.25 | 1.33 | 1.30 | -0.29 | -3.23 | 0.33 | 1.11 | -0.29 |
| PTF1A-Hs00603586_g1 | -0.26 | 1.32 | 1.29 | -0.30 | -3.20 | 0.33 | 1.10 | -0.30 |
| DES-Hs00157258_m1 | -3.50 | -0.70 | 0.96 | -1.97 | -1.48 | -0.01 | 7.07 | -0.38 |
| IAPP-Hs00169095_m1 | -1.57 | -0.46 | -1.21 | -3.03 | -0.07 | 3.51 | 4.28 | -1.45 |
| NPPA-Hs00383230_g1 | 0.57 | 2.15 | -2.01 | -4.96 | 0.62 | 1.16 | 1.93 | 0.53 |
| WT1-Hs00240913_m1 | 0.93 | 2.51 | -1.61 | -3.39 | -3.16 | 1.52 | 2.29 | 0.89 |
| GCG-Hs00174967_m1 | -0.12 | 1.46 | 1.43 | -0.16 | -0.07 | 0.46 | 1.24 | -4.24 |
| KRT1-Hs00196158_m1 | -0.13 | 1.45 | 1.42 | -0.17 | -0.08 | 0.46 | 1.23 | -4.18 |
| INS-Hs00355773_m1 | -0.25 | 1.33 | 1.30 | -0.29 | -0.20 | 0.34 | 1.11 | -3.33 |
| TAT-Hs00356930_m1 | 0.50 | 2.08 | 2.05 | 0.46 | -3.47 | 1.09 | 1.86 | -4.55 |
