# Supplementary material for: Specification of Region-Specific Neurons Including Forebrain Glutamatergic Neurons from Human Induced Pluripotent Stem Cells
Source: PLoS One. 2010 Jul 29;5(7):e11853. doi: 10.1371/journal.pone.0011853 (PMC2912324; doi:10.1371/journal.pone.0011853)
Supplement: Table S3 — (0.03 MB DOC) [file pone.0011853.s004.doc]

**Table S3. Recordings of electrophysiological parameters* on neurons differentiated from H9 hESC and TZ1 hiPSC in the basic neural induction condition without morphogens (see Figure 5)**

| **Cell type** | **H9** | **TZ1** |
| --- | --- | --- |
| Cells recorded | 30 | 40 |
| AP-detected cells | 23 | 30 |
| Averaged AP numbers | 2.0 ± 0.3 | 2.1 ± 0.2 |
| AP amplitude (mV) | 51.4 ± 2.1 | 49.6 ± 1.9 |
| TTX-sensitive Na^+^ currents (pA/pF) | 199.5 ± 10.5 (n = 6) | 195.6 ± 9.9 (n = 7) |
| 4-AP-senistive K^+^ currents (pA/pF) | 229.9 ± 15.2 (n = 6) | 220.1 ± 10.2 (n = 7) |
| TEA-sensitive K^+^ currents (pA/pF) | 204.8 ± 11.3 (n = 6) | 208 ± 12.4 (n = 7) |

*Based on Student *t* test, *P* > 0.05 for all the tested parameters between the two groups.
